# Outcomes of Intravascular Coronary Shockwave Lithotripsy: A Single-Center Tertiary Experience From the Middle East

**DOI:** 10.7759/cureus.105811

**Published:** 2026-03-25

**Authors:** Ebrahim K Al-Ebrahim, Azzam Alkhalifah, Tahir Mohamed

**Affiliations:** 1 Internal Medicine, King Faisal Specialist Hospital and Research Centre, Riyadh, SAU; 2 Interventional Adult Cardiology, King Faisal Specialist Hospital and Research Centre, Riyadh, SAU

**Keywords:** calcified coronary lesion, cto: chronic total occlusion, in-stent restenosis (isr), lithotripsy, shockwave

## Abstract

Heavily calcified coronary lesions present a significant challenge in percutaneous coronary intervention (PCI), often leading to complications such as stent underexpansion and malposition, which can negatively impact clinical outcomes. This retrospective study assesses the effectiveness of intravascular lithotripsy (IVL) in preparing calcified coronary lesions before stenting in native coronary lesions and in patients with in-stent restenosis (ISR). Data were collected from patients treated at King Faisal Specialist Hospital and Research Centre (KFSH&RC), Riyadh, between January 2019 and January 2025. Patients with coronary lesions exhibiting ≥70% stenosis requiring IVL were included. The primary objectives of the study are to assess key clinical outcomes, including repeat revascularization, target lesion revascularization, stroke, and major adverse cardiovascular events (MACE) post-IVL. Given the increasing use of novel lesion preparation techniques, this study aims to clarify whether IVL in native coronary or ISR offers effective and safe outcomes, potentially influencing treatment decisions for patients with severely calcified coronary lesions. In our study, IVL was demonstrated to be a safe and effective modality for the management of heavily calcified coronary lesions. Among the 45 enrolled patients, successful reperfusion was achieved in all cases, with 100% (45/45) attaining final thrombolysis in myocardial infarction (TIMI) 3 flow. No in-hospital MACE (0%) was reported.

## Introduction

The percutaneous treatment of heavily calcified coronary lesions continues to pose a significant challenge for interventional cardiologists, leading to an elevated risk of immediate complications, subsequent stent-related issues like underexpansion and malposition, and consequently, suboptimal clinical outcomes [1,2]. Fortunately, advancements in both traditional and novel specialized devices have improved the preparation of calcified lesions. Additionally, intracoronary imaging techniques play a crucial role in characterizing calcium distribution and optimizing the percutaneous treatment of these lesions. Nevertheless, addressing circumferential deep calcium plaques often necessitates a combination of two plaque-modification techniques: rotational atherectomy (RA) and intravascular lithotripsy (IVL). This innovative approach was initially introduced by our group as "RotaTripsy" [3]. Patients who have coronary disease with chronic total occlusion frequently manifest with severe calcifications; coronary intervention by the IVL system might be useful in maintaining and improving coronary perfusion [4,5]. Heavily calcified coronary lesions are considered challenging in interventional management and outcome, affecting the stent crossing, expansion, and placement. Classical interventional approach, including RA, results in ablation of the calcified lesion. Adverse events, including periprocedural myocardial infarction (MI), distal embolization, and dissection, are the main complications [6]. Advancements in coronary intervention resulted in a novel approach with IVL for heavily calcified coronary lesions before stenting. IVL appears to be a feasible novel approach for lesion preparation; however, further studies are needed to evaluate outcomes, including in patients with ISR [7]. The IVL system in the literature has been implicated with the incidence of ventricular ectopic “shocktopics” and asynchronous cardiac pacing; however, the incidence and risk factors for the phenomena inducing cardiac arrhythmia are lacking in the reported research [8]. Additionally, data regarding the outcomes of IVL technology in the Middle Eastern population are limited in the literature.

## Materials and methods

This is a retrospective study consisting of patients who underwent coronary IVL between January 2019 and January 2025 at the cardiac center of King Faisal Specialist Hospital and Research Centre (KFSH&RC) in Riyadh, Saudi Arabia. The subjects in this study were carefully selected from all consecutive patients with calcified lesions who underwent PCI and required additional lesion preparation with IVL, including the outcome and complications. The specified coronary vessels criteria for intervention should have more than 70% stenosis and be amenable to IVL. A heavily calcified coronary lesion was defined as the presence of severe coronary calcium on angiography, characterized by radiopaque densities without cardiac motion before contrast injection. Restenosis was defined as ≥50% luminal diameter reduction on follow-up angiography. The study included all patients who underwent the IVL procedure from 2019 to 2025 at KFSH&RC. Angiographic outcomes were assessed using conventional coronary angiography. Coronary flow was graded according to the thrombolysis in myocardial infarction (TIMI) flow classification, with post-procedural TIMI 3 flow considered indicative of optimal reperfusion. Follow-up was conducted through scheduled in-person clinical visits. Patients were assessed during physical visits for symptoms, adverse events, and clinical outcomes.

Inclusion criteria

Patients who underwent percutaneous coronary intervention (PCI) using IVL for severely calcified coronary lesions between January 2019 and January 2025 at KFSH&RC, Riyadh, Saudi Arabia, were included.

Exclusion criteria

Patients with severely calcified coronary lesions treated without IVL were excluded because the lesions were considered unsuitable for IVL or were managed using alternative calcium-modification strategies. This exclusion may introduce selection bias and limit the generalizability of the study findings.

The analyses were exploratory due to the limited sample size, which restricted the ability to perform adequately powered hypothesis-driven analyses. As a result, findings should be interpreted as descriptive and hypothesis-generating rather than confirmatory. Larger, prospective studies are needed to validate these observations.

Statistical analyses were performed using Stata version 18 (StataCorp LLC, College Station, TX). Continuous variables were summarized as median and interquartile range (IQR), and categorical variables as frequencies and percentages. Normality was assessed using the Shapiro-Wilk test. Categorical variables were compared using Fisher’s exact test when any expected cell count was less than 5.

To explore the association between patient characteristics and length of hospital stay (days), categorical variables were compared using the Mann-Whitney U test, while associations with continuous variables were assessed using Spearman’s rank correlation. Variables showing a potential association with length of stay, particularly gender and chronic kidney disease (CKD), were further evaluated using nonparametric kernel regression to estimate their effects on hospital length of stay, not adjusted for other confounders. A two-sided p-value < 0.05 was considered statistically significant.

## Results

Demographic and clinical profile

Table 1 shows the baseline characteristics of the study population (N = 45). Median age was 66 years (IQR: 58-71), and median BMI was 26.2 kg/m² (IQR: 23.9-29.8). The cohort included 31.1% females and 68.9% males. Diabetes was present in 86.7% of patients, hypertension in 77.8%, and a history of coronary artery disease in 93.3%. Heart failure was reported in 31.1% of patients, severe valvular heart disease in 11.1%, and malignancy in 2.2%.

**Table 1:** Baseline demographic and clinical characteristics (N = 45) PCI: Percutaneous coronary intervention; CABG: Coronary artery bypass graft; ESRD: End-stage renal disease; HCC: Hepatocellular carcinoma.

Variables	Frequency (%) or Median (IQR)
Age	66 (58-71)
BMI (kg/m²)	26.2 (23.9-29.8)
*Gender*	
Female	14 (31.1%)
Male	31 (68.9%)
*Smoking*	
No	35 (77.8%)
Yes	10 (22.2%)
*Diabetes mellites*	
No	6 (13.3%)
Yes	39 (86.7%)
*Dyslipidemia*	
No	22 (48.9%)
Yes	23 (51.1%)
*Hypertension*	
No	10 (22.2%)
Yes	35 (77.8%)
*History of coronary artery disease*	
No	3 (6.7%)
Yes	42 (93.3%)
*History of heart failure*	
No	31 (68.9%)
Yes	14 (31.1%)
*Previous myocardial infarction/acute coronary syndrome*	
No	13 (28.9%)
Yes	32 (71.1%)
*Family history of coronary artery disease*	
No	45 (100%)
Yes	0 (0%)
*Chronic kidney disease*	
No	24 (53.3%)
Yes	21 (46.7%)
*History of cerebrovascular accident*	
No	39 (86.7%)
Yes	6 (13.3%)
*Previous PCI*	
No	15 (33.3%)
Yes	30 (66.7%)
*Previous CABG*	
No	41 (91.1%)
Yes	4 (8.9%)
*History of peripheral arterial disease*	
No	39 (86.7%)
Yes	6 (13.3%)
*History of ventricular tachycardia/ventricular fibrillation*	
No	45 (100%)
Yes	0 (0%)
*History of atrial fibrillation*	
No	38 (84.4%)
Yes	7 (15.6%)
*History of the device*	
No	43 (95.6%)
Yes	2 (4.4%)
*History of severe valvular heart disease*	
No	40 (88.9%)
Yes	5 (11.1%)
*ESRD on dialysis*	
No	32 (71.1%)
Yes	13 (28.9%)
*History of COPD*	
No	42 (93.3%)
Yes	3 (6.7%)
*History of malignancy*	
No	44 (97.8%)
Yes (HCC)	1 (2.2%)

Patient presentation and baseline measurements

Baseline clinical presentation and laboratory measurements of the patients are summarized in Table 2. Classification was based on the primary clinical presentation, with secondary or minor features recorded separately and not included in the primary category. Unstable angina was the presentation in 42.2% of patients, non-ST elevation myocardial infarction (NSTEMI) in 17.8%, and heart failure in 11.1%. Median left ventricular ejection fraction (LVEF) before IVL was 50% (IQR 40-55%), and baseline hemoglobin was 118 g/L (IQR: 100-136).

**Table 2:** Clinical presentation (indication for intervention) (N = 45) NSTEMI: Non-ST elevation myocardial infarction; LVEF: Left ventricular ejection fraction; IVL: Intravascular lithotripsy.

Variables	Frequency (%) or Median (IQR)
*Stable angina*	
No	39 (86.7%)
Yes	6 (13.3%)
*Asymptomatic*	
No	38 (84.4%)
Yes	7 (15.6%)
*Unstable angina*	
No	26 (57.8%)
Yes	19 (42.2%)
*NSTEMI*	
No	37 (82.2%)
Yes	8 (17.8%)
*Heart failure*	
No	40 (88.9%)
Yes	5 (11.1%)
LVEF % (Before IVL)	50% (40%-55%)
Baseline hemoglobin, 100 g/L	118 (100-136)

Angiographic characteristics and procedural results

Table 3 shows the angiographic and operative data of the patients. Most lesions were located in the proximal segment (51.1%), followed by mid (28.9%) and ostial (13.3%) locations. Access was nearly equally divided between femoral (51.1%) and radial (48.9%) approaches. TIMI 3 flow was achieved in all patients (100%). Residual stenosis after IVL and subsequent stent implantation was 0%, reflecting the final post-stenting angiographic result (IQR: 0%-0%). Procedural success, defined as technical success without in-hospital MACE, was achieved in all patients, and no cases of failure to cross the wire for IVL were reported. No arrhythmias were reported during the periprocedural period. Continuous telemetry monitoring was used to detect any potential events.

**Table 3:** Angiographic and procedural data (N = 45) TIMI: Thrombolysis in myocardial infarction; MACE: Major adverse cardiovascular events.

Variables	Frequency (%) or Median (IQR)
*Lesion location*	
Distal	1 (2.2%)
Mid	13 (28.9%)
Ostial	6 (13.3%)
Proximal	23 (51.1%)
Proximal - Mid	2 (4.4%)
*Access (femoral and radial)*	
Femoral	23 (51.1%)
Radial	22 (48.9%)
*TIMI post-IVL*	
TIMI 3	45 (100%)
Residual stenosis % (post-IVL)	0 (0%-0%)
*Procedural success (technical success without in-hospital MACE)*	
No	0 (0%)
Yes	45 (100%)
*Attempted/Unable/Failure to cross wire for IVL*	
No	45 (100%)
Yes	0 (0%)

Table 4 shows procedure complications. One patient (2.2%) experienced both access site bleeding and access site hematoma, managed with a conservative approach. Septic shock occurred in one patient (2.2%). No other complications were reported. 

**Table 4:** Procedure complications (N = 45)

Variables	Frequency (%) or Median (IQR)
Access site bleeding with hemoglobin drop > 3 G	1 (2.2%)
Access site hematoma > 5 cm	1 (2.2%)
Coronary dissection	0 (0%)
Coronary perforation	0 (0%)
Pericardial tamponade	0 (0%)
Device entrapment	0 (0%)
Side branch occlusion	0 (0%)
Cardiogenic shock requiring inotropes	0 (0%)
Heart failure/acute pulmonary edema	0 (0%)
Cardiac arrest	0 (0%)
Arrhythmia	0 (0%)
Septic shock	1 (2.2%)
Other complication	0 (0%)

Table 5 summarizes in-hospital major adverse cardiac events (MACE). No events occurred. Median hospital stay was three days (IQR: 1-7). 

**Table 5:** In-hospital MACE (N = 45) URL: Upper range units; MACE: Major adverse cardiovascular events; MI: Myocardial infarction; PCI: Percutaneous coronary intervention; CABG: Coronary artery bypass graft.

Variables	Frequency (%) or Median (IQR)
Death (all-cause mortality)	0 (0%)
Cardiac death	0 (0%)
Non-cardiac death	0 (0%)
MI (troponin > 5 times URL or if baseline elevated > 20% increase)	0 (0%)
Stroke/cerebrovascular accident	0 (0%)
Target lesion revascularization (Yes, No, PCI, CABG)	0 (0%)
Target vessel revascularization other than IVL (Yes, No, PCI, CABG)	0 (0%)
Length of hospital stay in days	3 (1-7)

Table 6 summarizes one-year outcomes. All-cause mortality occurred in three patients (6.7%), including one cardiac death (2.2%) due to recurrent ventricular tachycardia with abrupt troponin rise leading to cardiac arrest and unsuccessful resuscitation as well as two non-cardiac deaths (4.4%). Stroke and angina were each reported in three patients (6.7%). Stroke events were confirmed based on clinical evaluation and supportive neuroimaging findings, and restenosis of the target lesion post-IVL was observed in 6.7% of patients. Median LVEF during follow-up was 50% (IQR: 45%-55%).

**Table 6:** One-year outcomes (N = 45) NYHA: New York Heart Association; LVEF: Left ventricular ejection fraction; IVL: Intravascular lithotripsy.

Variables	Frequency (%) or Median (IQR)
Death (all-cause mortality)	3 (6.7%)
Cardiac death	1 (2.2%)
Non-cardiac death	2 (4.4%)
Myocardial infarction	0 (0%)
Heart failure	0 (0%)
Stroke/cerebrovascular accident	3 (6.7%)
Angina	3 (6.7%)
Shortness of breath with NYHA class (I, II, III, IV)	1 (2.2%)
Restenosis of the target lesion post-IVL	3 (6.7%)
Restenosis requires revascularization (other than IVL)	1 (2.2%)
LVEF% during follow-up (last echo)	50% (45%-55%)

Impact of patient characteristics on hospital stay

Gender was significantly associated with length of hospital stay, with females having a longer median stay than males (7 days vs 1 day, p = 0.002) (Table 7). Chronic kidney disease showed a trend toward a longer stay, but this was not statistically significant (median 5 days vs 1.5 days, p = 0.071). End-stage renal disease (ESRD) on dialysis was not significantly associated with increased length of stay (p = 0.158).

**Table 7:** Association between categorical variables (gender, CKD, and ESRD) and length of hospital stay (days) ESRD: End-stage renal disease; CKD: Chronic kidney disease.

Variables		Length of Hospital Stay (Days), Median (IQR)	Mann–Whitney U Test	z-Value	p-Value
Gender	Female	7 (4–11)	96	3.053	0.002
	Male	1 (1–5)			
Chronic kidney disease	No	1.5 (1-6)	175	-1.815	0.071
	Yes	5 (2-8)			
ESRD on dialysis	No	2 (1-6.5)	153	-1.431	0.158
	Yes	5 (2-10)			

Outcomes in in-stent restenosis (ISR) versus native lesions

Table 8 presents a comparison of in-hospital and one-year outcomes between patients with in-stent and native vessels. Restenosis of the target lesion post-IVL occurred in one patient in the in-stent group and two patients in the native vessel group (p = 0.405). No significant differences were observed between the groups for length of hospital stay, all-cause mortality, cardiac death, non-cardiac death, stroke/CVA, angina, shortness of breath, target vessel revascularization, target lesion revascularization (IVL or other), or LVEF during follow-up, with all p-values > 0.05. These findings should be interpreted cautiously due to the small sample size.

**Table 8:** In-stent versus native vessel IVL outcomes LVEF: Left ventricular ejection fraction; IVL: Intravascular lithotripsy.

Variables	In-stent (n = 7)	Native (n = 38)	p-Value
Length of hospital stay in days	2 (1-7)	3 (1-7)	0.478
One-Year Outcomes			
*Death (all-cause mortality)*			
No	7	35	1.000
Yes	0	3	
*Cardiac death*			
No	7	37	1.000
Yes	0	1	
*Non-cardiac death*			
No	7	36	1.000
Yes	0	2	
*Stroke/cerebrovascular accident*			
No	7	35	1.000
Yes	0	3	
*Angina*			
No	6	36	0.405
Yes	1	2	
*Shortness of breath*			
No	7	37	1.000
Yes	0	1	
*Restenosis of the target lesion post-IVL*			
No	6	36	0.405
Yes	1	2	
LVEF% during follow-up	50% (45%-55%)	50% (45%-55%)	0.6943

Table 9 presents a comparison of the length of stay and LVEF % on follow-up between patients with ISR and those with native vessel lesions and demonstrates no significant difference.

**Table 9:** Comparison of length of hospital stay between patients with in-stent and native vessels LVEF: Left ventricular ejection fraction.

Variables		Length of Hospital Stay (Days), Median (IQR)	Mann–Whitney U Test	z-Value	p-Value
Target vessel	In stent	2 (1-7)	111	-0.709	0.478
	Native	3 (1-7)			
Target vessel	*LVEF% (one-year outcomes)*				
	In stent	50% (45%-55%)	121	0.393	0.6943
	Native	50% (40%-55%)			

Table 10 presents the relationship between age and length of hospital stay. No statistically significant association was identified, and hospitalization duration was comparable across age groups.

**Table 10:** Spearman correlation between age and length of hospital stay

Variable 1	Variable 2	N	Spearman ρ	p-Value
Age	Length of hospital stay	45	0.2410	0.1105

Table 11 presents the relationship between BMI and length of hospital stay. No statistically significant association was observed between the variable and hospitalization duration.

**Table 11:** Spearman correlation between BMI and length of hospital stay

Variable 1	Variable 2	N	Spearman ρ	p-Value
BMI	Length of hospital stay	45	0.0874	0.5664

## Discussion

Coronary artery calcification remains one of the most important anatomic drivers of suboptimal stent delivery and underexpansion and is consistently associated with higher rates of peri-procedural complications and late adverse events after PCI [9].

Lithotripsy was originally developed for the management of ureterorenal calculi and was subsequently adapted to the coronary setting as IVL, a balloon-based system that delivers acoustic shockwaves to disrupt calcified plaque, thereby facilitating improved stent expansion and luminal dimensions [9]. From a mechanistic standpoint, IVL induces calcium fractures that increase vessel compliance and facilitate luminal gain at relatively low pressures. The device is designed for use in severely calcified, stenotic de novo coronary lesions before stent placement. Each balloon catheter delivers up to 80 pulses at one pulse per second, administered as eight cycles of 10 pulses. After each 10-second cycle, the generator automatically stops and requires at least a 10-second pause before resuming. During this pause, the balloon must be fully deflated to avoid downstream coronary ischemia [10].

Intracoronary imaging studies using optical coherence tomography (OCT) or intravascular ultrasound (IVUS) have repeatedly demonstrated the presence of calcium fractures following IVL, along with improvements in minimal stent area and expansion, which are recognized correlates of better long-term outcomes after drug-eluting stent (DES) implantation [9]. IVL has demonstrated high device deliverability and favorable acute angiographic results in severely calcified de novo lesions [11]. In the pivotal Disrupt CAD III study, IVL facilitated successful stent implantation with high procedural success and low early adverse event rates, helping establish IVL as a standard calcium-modification option in modern cath labs [11].

Durability is a key question for any calcium-modification strategy because the clinical benefit depends on whether improved acute stent expansion translates into lower rates of restenosis and thrombosis [11]. Longer follow-up remains relatively limited, but available data are reassuring. A prespecified two-year report from Disrupt CAD III continued to show low rates of late stent thrombosis and repeat revascularization, supporting the hypothesis that mechanically optimized stent expansion after IVL is durable at least through intermediate follow-up [12].

Data from Japanese clinical experience provide additional insight into IVL performance across different populations and practice patterns. In the Japanese Disrupt CAD IV study, IVL was associated with high procedural success and acceptable short-term safety, supporting the generalizability of this technology beyond Western trial settings [13]. In the Spanish prospective multicenter REPLICA‑EPIC18 registry, IVL delivery was successful in nearly all cases, and short-term safety was favorable. Patients presenting with acute coronary syndrome (ACS) achieved similar angiographic success but showed a trend toward higher 30-day MACE compared with those presenting with chronic coronary syndrome, emphasizing the importance of clinical context when interpreting outcomes [14].

Future research priorities include randomized comparisons of IVL with atherectomy-based strategies in lesions with severe deep calcium; prospective studies focusing on ISR or underexpanded stents using standardized imaging criteria and clinical endpoints; and longer follow-up to determine whether imaging-proven expansion translates into durable reductions in target lesion revascularization (TLR) and stent thrombosis. In addition, as IVL use expands into ACS, complex bifurcations, and left main disease, registry infrastructure with core lab adjudication and granular procedural detail will remain important to understand best practices and mitigate complications [14]. Similarly, a multicenter European observational cohort with long-term follow-up reported very high procedural success and modest long-term rates of cardiac death, target vessel MI, and TLR. Notably, this cohort included a meaningful proportion of ISR cases and demonstrated that IVL can be integrated as either an upfront or bailout strategy, with occasional need for combined approaches such as “RotaTripsy” when lesions are extremely resistant [15].

Comparative effectiveness of atherectomy remains an area where the evidence base is still evolving. IVL offers technical simplicity (balloon‑based delivery) and reduced dependence on wire bias, while atherectomy (rotational/orbital) provides debulking and may be favored for very tight, uncrossable lesions where IVL balloon delivery is not possible. Real‑world registries show that combinations (e.g., RA followed by IVL - “RotaTripsy”) are sometimes required in the most resistant lesions, reflecting complementary rather than competing mechanisms [15]. In the BENELUX‑IVL registry analysis focusing on procedural complications, overall complications were uncommon, and only a small fraction was adjudicated as occurring immediately after IVL. However, patients who experienced complications had substantially higher one‑year MACE, largely driven by in‑hospital events, underscoring that complication avoidance remains clinically meaningful even if angiographic success can often be achieved with bailout measures [16].

IVL in ISR or underexpanded stents is most often considered when noncompliant high‑pressure balloons fail to expand the stent adequately, particularly when imaging shows heavy calcification behind the stent. Operators must balance the need for plaque modification against the risks of vessel injury, embolization, and stent deformation. Although IVL is delivered at low pressures, the presence of rigid stent scaffolds and prior interventions may predispose to perforation or dissection if the vessel is already compromised, an issue highlighted by registry analyses in which perforation, though infrequent, remains a recognized complication [16]. The role of imaging is even more critical when extending IVL beyond de novo calcified plaque into ISR, particularly ISR driven by stent underexpansion over persistent calcified plaque, neoatherosclerosis with calcified components, or calcified ring segments preventing further expansion [17]. Angiography alone may underestimate the depth and arc of calcium and the true mechanism of stent failure, and imaging can guide whether IVL is likely to be beneficial (e.g., calcium behind stent struts) versus scenarios where alternative therapies may be required (e.g., severe neointimal hyperplasia without calcified constraint) [17].

In‑stent applications represent a key “off‑label” frontier for IVL and are highly relevant to contemporary practice because resistant underexpanded stents remain one of the most challenging PCI failure modes. A contemporary systematic review and meta‑analysis evaluating IVL for underexpanded stents (often overlapping with ISR and stent thrombosis presentations) found high procedural success and low rates of major procedural complications, with imaging and quantitative coronary angiography demonstrating meaningful improvements in lumen and stent dimensions [17]. In our study, female sex was associated with a longer hospital length of stay. This finding may be related to differences in baseline clinical characteristics, comorbidity profiles, or overall recovery patterns between sexes. Variations in clinical presentation and in-hospital management may also contribute to prolonged hospitalization. These results are consistent with prior observations of sex-based differences in cardiovascular care and outcomes [18,19].

Limitation 

This study has several limitations that should be acknowledged. First, the relatively small sample size limits the statistical power and generalizability of the findings. Second, the single-center design may introduce selection and operator bias, potentially affecting external validity. In addition, outcomes were primarily based on angiographic assessment rather than intracoronary imaging modalities such as IVUS or OCT, which may provide more accurate evaluation of plaque modification and stent expansion. The lack of routine imaging guidance may have led to underestimation of residual disease or suboptimal expansion. Furthermore, the absence of a control group limits direct comparison with other calcium-modifying strategies. Larger, multicenter studies with longer follow-up and systematic use of intravascular imaging are warranted to validate these findings.

## Conclusions

IVL demonstrated a favorable safety profile and high procedural efficacy in the treatment of severely calcified coronary lesions. The technique consistently produced optimal angiographic outcomes, with final TIMI 3 flow achieved in all treated vessels, reflecting effective lesion preparation and stent expansion. Notably, IVL also showed favorable results in complex lesion subsets, including ISR, highlighting its utility in lesions that are typically challenging to treat with conventional calcium-modification strategies. The procedure allowed for predictable and controlled calcium disruption, facilitating optimal coronary perfusion while maintaining procedural safety. These findings suggest that IVL can improve both short- and long-term procedural outcomes, potentially reducing the risk of suboptimal stent deployment, restenosis, and future adverse cardiovascular events. Overall, this study supports IVL as a reliable, versatile, and effective strategy for managing heavily calcified coronary lesions, reinforcing its growing role in contemporary PCI and offering a valuable tool for operators managing high-risk and complex coronary anatomy.
